# {Bis[2-(diphenyl­phosphino)phen­yl] ether-κ^2^ *P*:*P*′}(dimethyl 2,2′-biphenyl-4,4′-dicarboxyl­ate-κ^2^ *N*:*N*′)copper(I) hexa­fluorido­phosphate acetonitrile solvate

**DOI:** 10.1107/S1600536809024982

**Published:** 2009-07-04

**Authors:** Ai-Guo Yu, Ming Zhang, Guang-Di Yang, Ling Ye, Yu-Guang Ma

**Affiliations:** a State Key Laboratory of Supramolecular Structure and Materials, College of Chemistry, Jilin University, Changchun 130012, People’s Republic of China

## Abstract

In the title compound, [Cu(C_14_H_12_N_2_O_4_)(C_36_H_28_OP_2_)]PF_6_·CH_3_CN, the  Cu(I) ion is coordinated by two N atoms from the dimethyl 2,2′-biphenyl-4,4′-dicarboxyl­ate ligand and two P atoms from the bis­[2-(diphenyl­phosphino)phen­yl] ether ligand in a distorted tetra­hedral environment. In the cation, the short distance of 3.870 (4) Å between the centroids of the benzene and phenyl rings suggests the existence of intra­molecular π–π inter­actions.

## Related literature

For background literature concerning Cu(I) complexes, see: Scaltrito *et al.* (2000 ▶). For related Cu(I) complexes, see: Ma *et al.* (1999 ▶).
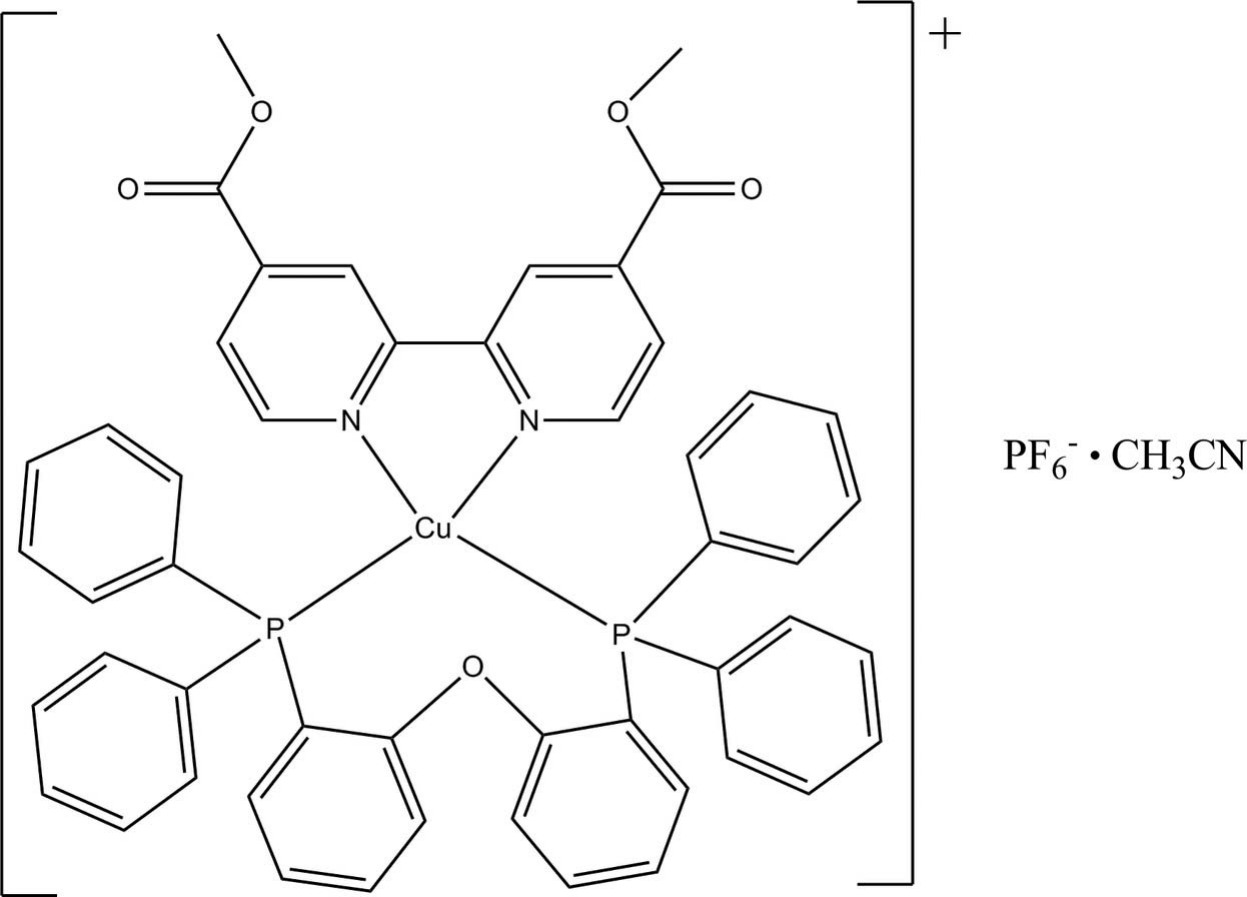


## Experimental

### 

#### Crystal data

[Cu(C_14_H_12_N_2_O_4_)(C_36_H_28_OP_2_)]PF_6_·C_2_H_3_N
*M* *_r_* = 1060.34
Triclinic, 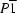

*a* = 10.7163 (16) Å
*b* = 11.9178 (12) Å
*c* = 19.879 (4) Å
α = 73.288 (5)°
β = 88.410 (5)°
γ = 79.863 (4)°
*V* = 2393.0 (6) Å^3^
*Z* = 2
Mo *K*α radiation
μ = 0.63 mm^−1^
*T* = 291 K
0.18 × 0.10 × 0.07 mm

#### Data collection

Rigaku R-AXIS RAPID diffractometer
Absorption correction: multi-scan (*ABSCOR*; Higashi, 1995 ▶) *T*_min_ = 0.895, *T*_max_ = 0.957
20165 measured reflections
10580 independent reflections
5125 reflections with *I* > 2σ(*I*)
*R*_int_ = 0.069

#### Refinement

*R*[*F* ^2^ > 2σ(*F* ^2^)] = 0.052
*wR*(*F* ^2^) = 0.141
*S* = 0.99
10580 reflections
632 parameters
H-atom parameters constrained
Δρ_max_ = 0.51 e Å^−3^
Δρ_min_ = −0.59 e Å^−3^

### 

Data collection: *RAPID-AUTO* (Rigaku, 1998 ▶); cell refinement: *RAPID-AUTO*; data reduction: *CrystalStructure* (Rigaku/MSC & Rigaku, 2002 ▶); program(s) used to solve structure: *SHELXS97* (Sheldrick, 2008 ▶); program(s) used to refine structure: *SHELXL97* (Sheldrick, 2008 ▶); molecular graphics: *PLATON* (Spek, 2009 ▶); software used to prepare material for publication: *SHELXL97*.

## Supplementary Material

Crystal structure: contains datablocks global, I. DOI: 10.1107/S1600536809024982/cv2564sup1.cif

Structure factors: contains datablocks I. DOI: 10.1107/S1600536809024982/cv2564Isup2.hkl

Additional supplementary materials:  crystallographic information; 3D view; checkCIF report

## Figures and Tables

**Fig. 1.**
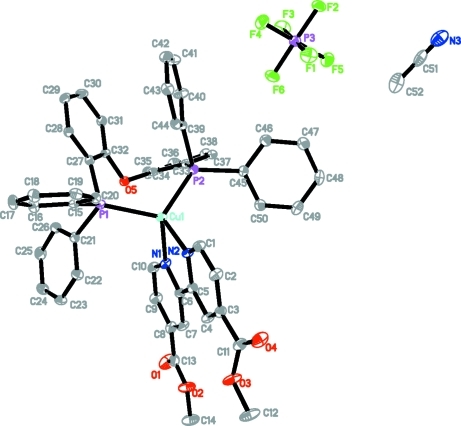
The content of asymmetric unit of the title compound, with the atom numbering. Displacement ellipsoids are drawn at the 30% probalility level. H atoms omitted for clarity.

## supplementary crystallographic information

### Comment

Recently, Ma *et al.* (1999) reported the first Cu(I) complex-based organic light-emitting diodes (OLEDs). Since that, Cu(I)complexes have received more attentions due to their available metal-to-ligand charge-transfer (MLCT) excited state and possible utilization in the OLEDs (Scaltrito *et al.*, 2000). Herein, we report the crystal structure of the title Cu(I) complex Dmfbpy-Cu-POP].PF_6_.CH_3_CN [Dmfbpy=4,7-dimethyl formate-2,2'-bipyridine, POP= bis[(2-diphenylphosphino)phenyl]ether], which have been proved to be a better material in the OLEDs applications.

In the title compound (Fig. 1), Cu1 is coordinated by two N atoms from one Dmfbpy ligand and two P atoms from one POP ligand, in a distorted tetrahedral configuration.

### Experimental

A mixture of [Cu(NCCH_3_)_4_].PF_6_.CH_3_CN (31 mg, 0.10 mmol) and bis- [2-(diphenylphosphino)phenyl]ether14 (54 mg, 0.10 mmol) in CH_2_Cl_2_ (20 ml) was stirred at room temperature for 2 h and then treated with a solution of 4, 7-dimethyl formate-2, 2'-bipyridine (27 mg, 0.10 mmol) in CH_2_Cl_2_ (5 ml). The reaction mixture was stirred for an additional 1 h and filtered, and the clear yellow filtrate was concentrated to *ca* 5 ml. Acetonitrile (about 5 ml) was added and the vapor diffusion of diethyl ether into the resulting solution. Orange crystals were obtained after about one week.

### Refinement

Carbon-bound H-atoms were placed in calculated positions (C—H 0.93 to 0.96) and were included in the refinement in the riding model with *U*_iso_(H) = 1.2 or 1.5 *U*_eq_(C).

### Crystal data

**Table:**

[Cu(C_14_H_12_N_2_O_4_)(C_36_H_28_OP_2_)]PF_6_·C_2_H_3_N	*Z* = 2
*M**_r_* = 1060.34	*F*(000) = 1088
Triclinic, *P*1	*D*_x_ = 1.472 Mg m^−^^3^
Hall symbol: -P 1	Mo *K*α radiation, λ = 0.71069 Å
*a* = 10.7163 (16) Å	Cell parameters from 9613 reflections
*b* = 11.9178 (12) Å	θ = 1.1–27.5°
*c* = 19.879 (4) Å	µ = 0.63 mm^−^^1^
α = 73.288 (5)°	*T* = 291 K
β = 88.410 (5)°	Block, red
γ = 79.863 (4)°	0.18 × 0.10 × 0.07 mm
*V* = 2393.0 (6) Å^3^	

### Data collection

**Table:**

Rigaku R-AXIS RAPID diffractometer	10580 independent reflections
Radiation source: fine-focus sealed tube	5125 reflections with *I* > 2σ(*I*)
graphite	*R*_int_ = 0.069
ω scans	θ_max_ = 27.5°, θ_min_ = 1.1°
Absorption correction: multi-scan (*ABSCOR*; Higashi, 1995)	*h* = 0→13
*T*_min_ = 0.895, *T*_max_ = 0.957	*k* = −14→15
20165 measured reflections	*l* = −25→25

### Refinement

**Table:**

Refinement on *F*^2^	Primary atom site location: structure-invariant direct methods
Least-squares matrix: full	Secondary atom site location: difference Fourier map
*R*[*F*^2^ > 2σ(*F*^2^)] = 0.052	Hydrogen site location: inferred from neighbouring sites
*wR*(*F*^2^) = 0.141	H-atom parameters constrained
*S* = 0.99	*w* = 1/[σ^2^(*F*_o_^2^) + (0.0437*P*)^2^] where *P* = (*F*_o_^2^ + 2*F*_c_^2^)/3
10580 reflections	(Δ/σ)_max_ < 0.001
632 parameters	Δρ_max_ = 0.51 e Å^−^^3^
0 restraints	Δρ_min_ = −0.59 e Å^−^^3^

### Special details

**Table:**

Experimental. (See detailed section in the paper)
Geometry. All e.s.d.'s (except the e.s.d. in the dihedral angle between two l.s. planes) are estimated using the full covariance matrix. The cell e.s.d.'s are taken into account individually in the estimation of e.s.d.'s in distances, angles and torsion angles; correlations between e.s.d.'s in cell parameters are only used when they are defined by crystal symmetry. An approximate (isotropic) treatment of cell e.s.d.'s is used for estimating e.s.d.'s involving l.s. planes.
Refinement. Refinement of *F*^2^ against ALL reflections. The weighted *R*-factor *wR* and goodness of fit *S* are based on *F*^2^, conventional *R*-factors *R* are based on *F*, with *F* set to zero for negative *F*^2^. The threshold expression of *F*^2^ > σ(*F*^2^) is used only for calculating *R*-factors(gt) *etc*. and is not relevant to the choice of reflections for refinement. *R*-factors based on *F*^2^ are statistically about twice as large as those based on *F*, and *R*- factors based on ALL data will be even larger.

### Fractional atomic coordinates and isotropic or equivalent isotropic displacement parameters (Å^2^)

**Table:**

	*x*	*y*	*z*	*U*_iso_*/*U*_eq_	
C51	0.6814 (7)	0.7193 (6)	0.3887 (3)	0.0479 (17)	
Cu1	0.06564 (6)	0.10575 (5)	0.25353 (3)	0.02056 (16)	
F1	0.5125 (3)	0.6034 (3)	0.22930 (18)	0.0521 (9)	
F2	0.6796 (3)	0.6833 (2)	0.17910 (15)	0.0407 (8)	
F3	0.7751 (3)	0.5018 (3)	0.17083 (18)	0.0566 (10)	
F4	0.5795 (4)	0.5795 (3)	0.12452 (16)	0.0574 (10)	
F5	0.7092 (3)	0.5259 (3)	0.27506 (15)	0.0486 (9)	
F6	0.6081 (3)	0.4229 (2)	0.22121 (16)	0.0462 (9)	
P1	0.00627 (12)	0.06619 (10)	0.15539 (6)	0.0197 (3)	
P2	0.22917 (12)	0.20575 (11)	0.23499 (6)	0.0197 (3)	
P3	0.64319 (14)	0.55331 (12)	0.19925 (7)	0.0295 (3)	
N1	0.1019 (4)	−0.0566 (3)	0.33146 (19)	0.0212 (9)	
N2	−0.0701 (4)	0.1398 (3)	0.32392 (18)	0.0210 (9)	
N3	0.7574 (6)	0.7774 (5)	0.3895 (3)	0.0604 (16)	
O1	0.2117 (4)	−0.4602 (3)	0.5059 (2)	0.0562 (13)	
O2	0.0392 (4)	−0.3704 (3)	0.54642 (18)	0.0407 (10)	
O3	−0.3613 (4)	0.0578 (3)	0.52117 (18)	0.0419 (10)	
O4	−0.4075 (4)	0.2579 (3)	0.48518 (19)	0.0407 (10)	
O5	0.2759 (3)	0.0032 (3)	0.16853 (15)	0.0213 (7)	
C1	−0.1549 (5)	0.2395 (4)	0.3183 (2)	0.0255 (12)	
H1A	−0.1516	0.3044	0.2792	0.031*	
C2	−0.2474 (5)	0.2518 (4)	0.3673 (2)	0.0273 (12)	
H2A	−0.3041	0.3228	0.3614	0.033*	
C3	−0.2522 (5)	0.1548 (4)	0.4253 (3)	0.0272 (12)	
C4	−0.1664 (5)	0.0515 (4)	0.4317 (2)	0.0265 (12)	
H4A	−0.1694	−0.0144	0.4702	0.032*	
C5	−0.0747 (4)	0.0450 (4)	0.3809 (2)	0.0213 (11)	
C6	0.0201 (4)	−0.0628 (4)	0.3854 (2)	0.0217 (11)	
C7	0.0276 (5)	−0.1665 (4)	0.4413 (2)	0.0264 (12)	
H7A	−0.0276	−0.1695	0.4785	0.032*	
C8	0.1182 (5)	−0.2651 (4)	0.4408 (3)	0.0270 (12)	
C9	0.1982 (5)	−0.2586 (4)	0.3845 (3)	0.0294 (12)	
H9A	0.2591	−0.3237	0.3828	0.035*	
C10	0.1858 (5)	−0.1539 (4)	0.3314 (3)	0.0263 (12)	
H10A	0.2387	−0.1504	0.2932	0.032*	
C11	−0.3490 (5)	0.1656 (5)	0.4802 (3)	0.0305 (13)	
C12	−0.4542 (5)	0.0575 (5)	0.5763 (3)	0.0490 (17)	
H12A	−0.4567	−0.0231	0.6032	0.073*	
H12B	−0.5363	0.0947	0.5553	0.073*	
H12C	−0.4306	0.1006	0.6065	0.073*	
C13	0.1292 (5)	−0.3773 (4)	0.5004 (3)	0.0314 (13)	
C14	0.0418 (6)	−0.4757 (5)	0.6060 (3)	0.0492 (17)	
H14A	−0.0268	−0.4619	0.6363	0.074*	
H14B	0.1209	−0.4926	0.6314	0.074*	
H14C	0.0329	−0.5422	0.5894	0.074*	
C15	−0.1493 (4)	0.1290 (4)	0.1134 (2)	0.0203 (10)	
C16	−0.2012 (5)	0.0822 (4)	0.0659 (3)	0.0271 (12)	
H16A	−0.1584	0.0129	0.0575	0.032*	
C17	−0.3145 (5)	0.1373 (4)	0.0318 (3)	0.0302 (12)	
H17A	−0.3479	0.1057	0.0001	0.036*	
C18	−0.3800 (5)	0.2417 (4)	0.0447 (3)	0.0261 (12)	
H18A	−0.4562	0.2798	0.0208	0.031*	
C19	−0.3319 (5)	0.2883 (4)	0.0927 (3)	0.0247 (11)	
H19A	−0.3759	0.3566	0.1019	0.030*	
C20	−0.2162 (4)	0.2311 (4)	0.1269 (2)	0.0206 (11)	
H20A	−0.1833	0.2617	0.1593	0.025*	
C21	0.0181 (4)	−0.0960 (4)	0.1721 (2)	0.0212 (11)	
C22	−0.0564 (5)	−0.1527 (4)	0.2250 (3)	0.0274 (12)	
H22A	−0.1118	−0.1081	0.2483	0.033*	
C23	−0.0490 (5)	−0.2751 (4)	0.2433 (3)	0.0305 (13)	
H23A	−0.1009	−0.3120	0.2778	0.037*	
C24	0.0349 (5)	−0.3415 (4)	0.2104 (3)	0.0351 (14)	
H24A	0.0398	−0.4236	0.2230	0.042*	
C25	0.1122 (5)	−0.2881 (4)	0.1590 (3)	0.0338 (13)	
H25A	0.1704	−0.3339	0.1377	0.041*	
C26	0.1023 (5)	−0.1651 (4)	0.1394 (3)	0.0264 (12)	
H26A	0.1526	−0.1286	0.1039	0.032*	
C27	0.1159 (5)	0.1071 (4)	0.0842 (2)	0.0198 (11)	
C28	0.0783 (5)	0.1778 (4)	0.0161 (2)	0.0210 (11)	
H28A	−0.0072	0.2015	0.0034	0.025*	
C29	0.1711 (5)	0.2126 (4)	−0.0326 (2)	0.0256 (12)	
H29A	0.1464	0.2599	−0.0779	0.031*	
C30	0.2969 (5)	0.1785 (4)	−0.0150 (3)	0.0294 (12)	
H30A	0.3569	0.2026	−0.0483	0.035*	
C31	0.3356 (5)	0.1087 (4)	0.0516 (2)	0.0224 (11)	
H31A	0.4214	0.0848	0.0637	0.027*	
C32	0.2443 (4)	0.0747 (4)	0.1003 (2)	0.0187 (10)	
C33	0.3828 (4)	0.1127 (4)	0.2280 (2)	0.0203 (11)	
C34	0.3867 (4)	0.0176 (4)	0.1984 (2)	0.0212 (11)	
C35	0.4954 (5)	−0.0657 (4)	0.2003 (3)	0.0290 (12)	
H35A	0.4948	−0.1295	0.1823	0.035*	
C36	0.6040 (5)	−0.0515 (5)	0.2294 (3)	0.0333 (13)	
H36A	0.6776	−0.1069	0.2311	0.040*	
C37	0.6069 (5)	0.0432 (4)	0.2564 (3)	0.0302 (12)	
H37A	0.6824	0.0524	0.2743	0.036*	
C38	0.4964 (5)	0.1241 (4)	0.2564 (2)	0.0250 (11)	
H38A	0.4980	0.1865	0.2756	0.030*	
C39	0.2108 (5)	0.3287 (4)	0.1550 (2)	0.0213 (11)	
C40	0.3114 (5)	0.3613 (4)	0.1123 (3)	0.0315 (13)	
H40A	0.3930	0.3185	0.1238	0.038*	
C41	0.2893 (5)	0.4575 (4)	0.0528 (3)	0.0365 (14)	
H41A	0.3562	0.4788	0.0240	0.044*	
C42	0.1698 (5)	0.5215 (4)	0.0363 (3)	0.0351 (14)	
H42A	0.1561	0.5863	−0.0038	0.042*	
C43	0.0689 (5)	0.4908 (4)	0.0784 (3)	0.0288 (12)	
H43A	−0.0121	0.5350	0.0669	0.035*	
C44	0.0894 (5)	0.3943 (4)	0.1376 (2)	0.0228 (11)	
H44A	0.0219	0.3731	0.1660	0.027*	
C45	0.2621 (4)	0.2768 (4)	0.3017 (2)	0.0213 (11)	
C46	0.3170 (5)	0.3784 (4)	0.2882 (3)	0.0322 (13)	
H46A	0.3342	0.4174	0.2421	0.039*	
C47	0.3466 (5)	0.4232 (5)	0.3421 (3)	0.0371 (14)	
H47A	0.3832	0.4912	0.3317	0.044*	
C48	0.3217 (5)	0.3667 (5)	0.4107 (3)	0.0353 (13)	
H48A	0.3416	0.3958	0.4470	0.042*	
C49	0.2667 (5)	0.2661 (5)	0.4247 (3)	0.0356 (13)	
H49A	0.2487	0.2281	0.4708	0.043*	
C50	0.2382 (5)	0.2212 (4)	0.3718 (3)	0.0292 (12)	
H50A	0.2024	0.1527	0.3828	0.035*	
C52	0.5815 (6)	0.6510 (6)	0.3872 (3)	0.0615 (19)	
H52A	0.5195	0.6627	0.4215	0.092*	
H52B	0.6176	0.5680	0.3979	0.092*	
H52C	0.5417	0.6773	0.3414	0.092*	

### Atomic displacement parameters (Å^2^)

**Table:**

	*U*^11^	*U*^22^	*U*^33^	*U*^12^	*U*^13^	*U*^23^
C51	0.045 (4)	0.065 (5)	0.028 (3)	0.016 (4)	0.001 (3)	−0.020 (3)
Cu1	0.0201 (3)	0.0210 (3)	0.0187 (3)	−0.0018 (3)	0.0035 (3)	−0.0041 (3)
F1	0.039 (2)	0.050 (2)	0.064 (2)	0.0011 (17)	0.0155 (18)	−0.0172 (18)
F2	0.051 (2)	0.0241 (15)	0.0433 (19)	−0.0080 (15)	0.0036 (16)	−0.0033 (14)
F3	0.050 (2)	0.049 (2)	0.073 (3)	−0.0006 (17)	0.026 (2)	−0.0271 (19)
F4	0.085 (3)	0.050 (2)	0.0334 (19)	−0.011 (2)	−0.0143 (19)	−0.0051 (16)
F5	0.068 (3)	0.0371 (17)	0.0332 (18)	0.0031 (17)	−0.0140 (17)	−0.0039 (15)
F6	0.058 (2)	0.0294 (16)	0.051 (2)	−0.0155 (16)	0.0066 (18)	−0.0073 (15)
P1	0.0176 (7)	0.0206 (6)	0.0192 (6)	−0.0010 (5)	0.0022 (5)	−0.0050 (5)
P2	0.0185 (7)	0.0219 (6)	0.0180 (6)	−0.0022 (5)	0.0019 (5)	−0.0057 (5)
P3	0.0339 (9)	0.0251 (7)	0.0272 (7)	−0.0016 (6)	0.0035 (7)	−0.0061 (6)
N1	0.019 (2)	0.023 (2)	0.017 (2)	0.0035 (18)	0.0004 (17)	−0.0040 (17)
N2	0.022 (2)	0.020 (2)	0.017 (2)	−0.0009 (18)	0.0035 (18)	−0.0023 (17)
N3	0.056 (4)	0.068 (4)	0.042 (3)	0.013 (3)	−0.001 (3)	−0.006 (3)
O1	0.079 (4)	0.028 (2)	0.042 (2)	0.025 (2)	0.007 (2)	0.0002 (19)
O2	0.038 (2)	0.032 (2)	0.033 (2)	0.0030 (18)	0.0040 (19)	0.0133 (18)
O3	0.045 (3)	0.037 (2)	0.031 (2)	0.0039 (19)	0.0225 (19)	0.0013 (18)
O4	0.043 (3)	0.036 (2)	0.035 (2)	0.0113 (19)	0.0118 (19)	−0.0103 (18)
O5	0.0191 (19)	0.0252 (17)	0.0189 (17)	−0.0047 (15)	0.0013 (15)	−0.0048 (15)
C1	0.028 (3)	0.022 (3)	0.021 (3)	0.001 (2)	0.001 (2)	−0.001 (2)
C2	0.026 (3)	0.023 (3)	0.026 (3)	0.008 (2)	0.000 (2)	−0.004 (2)
C3	0.030 (3)	0.028 (3)	0.025 (3)	−0.004 (2)	0.009 (2)	−0.012 (2)
C4	0.026 (3)	0.028 (3)	0.020 (3)	0.000 (2)	0.002 (2)	−0.001 (2)
C5	0.020 (3)	0.023 (2)	0.019 (2)	−0.001 (2)	0.000 (2)	−0.005 (2)
C6	0.022 (3)	0.021 (2)	0.022 (3)	−0.004 (2)	0.001 (2)	−0.005 (2)
C7	0.033 (3)	0.027 (3)	0.015 (2)	−0.003 (2)	0.003 (2)	−0.001 (2)
C8	0.029 (3)	0.023 (3)	0.024 (3)	0.005 (2)	−0.008 (2)	−0.003 (2)
C9	0.031 (3)	0.018 (2)	0.031 (3)	0.008 (2)	−0.001 (3)	−0.002 (2)
C10	0.023 (3)	0.028 (3)	0.026 (3)	0.002 (2)	0.004 (2)	−0.009 (2)
C11	0.025 (3)	0.039 (3)	0.025 (3)	−0.002 (3)	0.004 (2)	−0.008 (3)
C12	0.040 (4)	0.058 (4)	0.036 (3)	0.003 (3)	0.023 (3)	−0.002 (3)
C13	0.041 (4)	0.028 (3)	0.026 (3)	−0.007 (3)	−0.003 (3)	−0.008 (2)
C14	0.054 (4)	0.042 (3)	0.034 (3)	−0.004 (3)	0.002 (3)	0.015 (3)
C15	0.020 (3)	0.019 (2)	0.018 (2)	−0.003 (2)	0.004 (2)	−0.001 (2)
C16	0.021 (3)	0.030 (3)	0.034 (3)	−0.001 (2)	0.003 (2)	−0.018 (2)
C17	0.023 (3)	0.040 (3)	0.034 (3)	−0.008 (3)	−0.003 (2)	−0.019 (3)
C18	0.012 (3)	0.034 (3)	0.033 (3)	−0.003 (2)	0.000 (2)	−0.010 (2)
C19	0.019 (3)	0.018 (2)	0.033 (3)	0.000 (2)	0.003 (2)	−0.003 (2)
C20	0.021 (3)	0.020 (2)	0.021 (2)	−0.005 (2)	0.001 (2)	−0.006 (2)
C21	0.021 (3)	0.019 (2)	0.023 (3)	−0.002 (2)	−0.003 (2)	−0.005 (2)
C22	0.024 (3)	0.027 (3)	0.032 (3)	−0.004 (2)	0.006 (2)	−0.009 (2)
C23	0.038 (3)	0.022 (3)	0.028 (3)	−0.011 (2)	0.001 (3)	0.000 (2)
C24	0.048 (4)	0.020 (3)	0.036 (3)	−0.009 (3)	−0.002 (3)	−0.004 (2)
C25	0.041 (4)	0.025 (3)	0.037 (3)	−0.001 (3)	0.003 (3)	−0.016 (3)
C26	0.028 (3)	0.028 (3)	0.027 (3)	−0.007 (2)	0.007 (2)	−0.012 (2)
C27	0.027 (3)	0.012 (2)	0.020 (2)	−0.001 (2)	0.003 (2)	−0.007 (2)
C28	0.019 (3)	0.023 (2)	0.022 (3)	−0.003 (2)	0.000 (2)	−0.008 (2)
C29	0.033 (3)	0.023 (3)	0.018 (2)	−0.003 (2)	0.002 (2)	−0.004 (2)
C30	0.031 (3)	0.031 (3)	0.027 (3)	−0.011 (2)	0.013 (3)	−0.008 (2)
C31	0.017 (3)	0.027 (3)	0.023 (3)	−0.004 (2)	0.001 (2)	−0.008 (2)
C32	0.020 (3)	0.018 (2)	0.017 (2)	−0.003 (2)	0.000 (2)	−0.003 (2)
C33	0.017 (3)	0.021 (2)	0.019 (2)	−0.001 (2)	0.001 (2)	−0.002 (2)
C34	0.020 (3)	0.022 (2)	0.018 (2)	−0.004 (2)	0.002 (2)	0.000 (2)
C35	0.024 (3)	0.029 (3)	0.034 (3)	0.002 (2)	0.002 (2)	−0.013 (2)
C36	0.021 (3)	0.035 (3)	0.039 (3)	0.004 (2)	−0.001 (3)	−0.008 (3)
C37	0.015 (3)	0.042 (3)	0.030 (3)	−0.001 (2)	−0.006 (2)	−0.006 (3)
C38	0.028 (3)	0.025 (3)	0.021 (3)	−0.008 (2)	0.004 (2)	−0.004 (2)
C39	0.025 (3)	0.020 (2)	0.021 (2)	−0.004 (2)	0.002 (2)	−0.008 (2)
C40	0.026 (3)	0.028 (3)	0.030 (3)	0.005 (2)	0.006 (2)	0.003 (2)
C41	0.035 (4)	0.027 (3)	0.039 (3)	−0.001 (3)	0.010 (3)	−0.001 (3)
C42	0.050 (4)	0.023 (3)	0.028 (3)	−0.005 (3)	0.001 (3)	−0.002 (2)
C43	0.025 (3)	0.021 (3)	0.036 (3)	0.004 (2)	−0.010 (3)	−0.005 (2)
C44	0.020 (3)	0.023 (2)	0.026 (3)	−0.004 (2)	0.000 (2)	−0.007 (2)
C45	0.016 (3)	0.025 (3)	0.023 (3)	−0.001 (2)	0.000 (2)	−0.009 (2)
C46	0.034 (3)	0.033 (3)	0.032 (3)	−0.008 (3)	0.006 (3)	−0.011 (3)
C47	0.043 (4)	0.033 (3)	0.039 (3)	−0.009 (3)	0.003 (3)	−0.013 (3)
C48	0.036 (3)	0.042 (3)	0.034 (3)	0.000 (3)	−0.006 (3)	−0.022 (3)
C49	0.035 (3)	0.045 (3)	0.028 (3)	−0.001 (3)	0.001 (3)	−0.016 (3)
C50	0.030 (3)	0.029 (3)	0.029 (3)	−0.006 (2)	0.002 (2)	−0.009 (2)
C52	0.044 (4)	0.085 (5)	0.057 (4)	0.011 (4)	−0.006 (4)	−0.034 (4)

### Geometric parameters (Å, °)

**Table:**

C51—N3	1.162 (8)	C18—H18A	0.9300
C51—C52	1.460 (9)	C19—C20	1.395 (6)
Cu1—N2	2.060 (4)	C19—H19A	0.9300
Cu1—N1	2.083 (4)	C20—H20A	0.9300
Cu1—P2	2.2526 (14)	C21—C26	1.389 (6)
Cu1—P1	2.2665 (14)	C21—C22	1.390 (6)
F1—P3	1.593 (3)	C22—C23	1.386 (6)
F2—P3	1.600 (3)	C22—H22A	0.9300
F3—P3	1.595 (3)	C23—C24	1.369 (7)
F4—P3	1.576 (3)	C23—H23A	0.9300
F5—P3	1.604 (3)	C24—C25	1.378 (7)
F6—P3	1.599 (3)	C24—H24A	0.9300
P1—C15	1.820 (5)	C25—C26	1.389 (6)
P1—C27	1.825 (4)	C25—H25A	0.9300
P1—C21	1.846 (4)	C26—H26A	0.9300
P2—C39	1.814 (5)	C27—C32	1.383 (6)
P2—C45	1.837 (5)	C27—C28	1.400 (6)
P2—C33	1.842 (5)	C28—C29	1.400 (6)
N1—C10	1.337 (6)	C28—H28A	0.9300
N1—C6	1.359 (5)	C29—C30	1.362 (7)
N2—C1	1.341 (6)	C29—H29A	0.9300
N2—C5	1.358 (5)	C30—C31	1.377 (6)
O1—C13	1.186 (6)	C30—H30A	0.9300
O2—C13	1.319 (6)	C31—C32	1.385 (6)
O2—C14	1.453 (5)	C31—H31A	0.9300
O3—C11	1.335 (6)	C33—C38	1.401 (7)
O3—C12	1.459 (6)	C33—C34	1.413 (6)
O4—C11	1.195 (6)	C34—C35	1.385 (6)
O5—C32	1.393 (5)	C35—C36	1.370 (7)
O5—C34	1.401 (5)	C35—H35A	0.9300
C1—C2	1.390 (6)	C36—C37	1.387 (7)
C1—H1A	0.9300	C36—H36A	0.9300
C2—C3	1.386 (6)	C37—C38	1.390 (7)
C2—H2A	0.9300	C37—H37A	0.9300
C3—C4	1.376 (6)	C38—H38A	0.9300
C3—C11	1.501 (6)	C39—C44	1.391 (6)
C4—C5	1.399 (6)	C39—C40	1.392 (6)
C4—H4A	0.9300	C40—C41	1.381 (7)
C5—C6	1.473 (6)	C40—H40A	0.9300
C6—C7	1.396 (6)	C41—C42	1.366 (7)
C7—C8	1.389 (6)	C41—H41A	0.9300
C7—H7A	0.9300	C42—C43	1.385 (7)
C8—C9	1.383 (7)	C42—H42A	0.9300
C8—C13	1.500 (7)	C43—C44	1.380 (6)
C9—C10	1.372 (6)	C43—H43A	0.9300
C9—H9A	0.9300	C44—H44A	0.9300
C10—H10A	0.9300	C45—C46	1.393 (6)
C12—H12A	0.9600	C45—C50	1.399 (6)
C12—H12B	0.9600	C46—C47	1.393 (7)
C12—H12C	0.9600	C46—H46A	0.9300
C14—H14A	0.9600	C47—C48	1.379 (7)
C14—H14B	0.9600	C47—H47A	0.9300
C14—H14C	0.9600	C48—C49	1.382 (7)
C15—C20	1.393 (6)	C48—H48A	0.9300
C15—C16	1.397 (7)	C49—C50	1.372 (7)
C16—C17	1.369 (7)	C49—H49A	0.9300
C16—H16A	0.9300	C50—H50A	0.9300
C17—C18	1.404 (7)	C52—H52A	0.9600
C17—H17A	0.9300	C52—H52B	0.9600
C18—C19	1.385 (7)	C52—H52C	0.9600
			
N3—C51—C52	177.5 (7)	C18—C19—C20	118.9 (4)
N2—Cu1—N1	80.69 (14)	C18—C19—H19A	120.5
N2—Cu1—P2	118.87 (11)	C20—C19—H19A	120.5
N1—Cu1—P2	113.94 (11)	C15—C20—C19	121.0 (4)
N2—Cu1—P1	119.29 (12)	C15—C20—H20A	119.5
N1—Cu1—P1	106.56 (11)	C19—C20—H20A	119.5
P2—Cu1—P1	112.38 (5)	C26—C21—C22	118.4 (4)
C15—P1—C27	103.6 (2)	C26—C21—P1	124.5 (4)
C15—P1—C21	104.4 (2)	C22—C21—P1	116.8 (3)
C27—P1—C21	104.0 (2)	C23—C22—C21	120.7 (5)
C15—P1—Cu1	122.05 (16)	C23—C22—H22A	119.7
C27—P1—Cu1	111.41 (16)	C21—C22—H22A	119.7
C21—P1—Cu1	109.74 (16)	C24—C23—C22	119.8 (5)
C39—P2—C45	102.6 (2)	C24—C23—H23A	120.1
C39—P2—C33	105.4 (2)	C22—C23—H23A	120.1
C45—P2—C33	103.0 (2)	C23—C24—C25	120.8 (4)
C39—P2—Cu1	113.39 (16)	C23—C24—H24A	119.6
C45—P2—Cu1	117.33 (15)	C25—C24—H24A	119.6
C33—P2—Cu1	113.64 (15)	C24—C25—C26	119.3 (5)
F4—P3—F1	91.1 (2)	C24—C25—H25A	120.4
F4—P3—F3	90.0 (2)	C26—C25—H25A	120.4
F1—P3—F3	178.8 (2)	C21—C26—C25	120.9 (4)
F4—P3—F6	90.00 (18)	C21—C26—H26A	119.5
F1—P3—F6	90.30 (18)	C25—C26—H26A	119.5
F3—P3—F6	89.34 (17)	C32—C27—C28	117.9 (4)
F4—P3—F2	91.34 (18)	C32—C27—P1	117.7 (3)
F1—P3—F2	89.61 (17)	C28—C27—P1	124.1 (4)
F3—P3—F2	90.72 (17)	C27—C28—C29	119.2 (5)
F6—P3—F2	178.66 (19)	C27—C28—H28A	120.4
F4—P3—F5	179.4 (2)	C29—C28—H28A	120.4
F1—P3—F5	89.45 (19)	C30—C29—C28	121.2 (4)
F3—P3—F5	89.4 (2)	C30—C29—H29A	119.4
F6—P3—F5	89.90 (17)	C28—C29—H29A	119.4
F2—P3—F5	88.76 (17)	C29—C30—C31	120.4 (4)
C10—N1—C6	118.5 (4)	C29—C30—H30A	119.8
C10—N1—Cu1	128.4 (3)	C31—C30—H30A	119.8
C6—N1—Cu1	112.9 (3)	C30—C31—C32	118.8 (5)
C1—N2—C5	118.2 (4)	C30—C31—H31A	120.6
C1—N2—Cu1	128.3 (3)	C32—C31—H31A	120.6
C5—N2—Cu1	113.4 (3)	C27—C32—C31	122.5 (4)
C13—O2—C14	115.8 (4)	C27—C32—O5	115.3 (4)
C11—O3—C12	115.0 (4)	C31—C32—O5	122.2 (4)
C32—O5—C34	116.3 (3)	C38—C33—C34	117.2 (4)
N2—C1—C2	123.9 (4)	C38—C33—P2	123.2 (4)
N2—C1—H1A	118.0	C34—C33—P2	119.4 (4)
C2—C1—H1A	118.0	C35—C34—O5	118.2 (4)
C3—C2—C1	117.8 (4)	C35—C34—C33	122.4 (5)
C3—C2—H2A	121.1	O5—C34—C33	119.4 (4)
C1—C2—H2A	121.1	C36—C35—C34	118.2 (5)
C4—C3—C2	119.0 (4)	C36—C35—H35A	120.9
C4—C3—C11	121.7 (4)	C34—C35—H35A	120.9
C2—C3—C11	119.3 (5)	C35—C36—C37	121.7 (5)
C3—C4—C5	120.5 (4)	C35—C36—H36A	119.1
C3—C4—H4A	119.7	C37—C36—H36A	119.1
C5—C4—H4A	119.7	C36—C37—C38	119.7 (5)
N2—C5—C4	120.5 (4)	C36—C37—H37A	120.2
N2—C5—C6	116.6 (4)	C38—C37—H37A	120.2
C4—C5—C6	122.9 (4)	C37—C38—C33	120.7 (5)
N1—C6—C7	120.8 (4)	C37—C38—H38A	119.7
N1—C6—C5	116.2 (4)	C33—C38—H38A	119.7
C7—C6—C5	122.9 (4)	C44—C39—C40	119.6 (4)
C8—C7—C6	119.3 (4)	C44—C39—P2	116.9 (3)
C8—C7—H7A	120.3	C40—C39—P2	123.5 (4)
C6—C7—H7A	120.3	C41—C40—C39	119.7 (5)
C9—C8—C7	119.2 (4)	C41—C40—H40A	120.2
C9—C8—C13	120.3 (4)	C39—C40—H40A	120.2
C7—C8—C13	120.5 (5)	C42—C41—C40	120.3 (5)
C10—C9—C8	118.5 (4)	C42—C41—H41A	119.8
C10—C9—H9A	120.8	C40—C41—H41A	119.8
C8—C9—H9A	120.8	C41—C42—C43	120.7 (5)
N1—C10—C9	123.6 (4)	C41—C42—H42A	119.6
N1—C10—H10A	118.2	C43—C42—H42A	119.6
C9—C10—H10A	118.2	C44—C43—C42	119.6 (5)
O4—C11—O3	125.5 (4)	C44—C43—H43A	120.2
O4—C11—C3	124.4 (5)	C42—C43—H43A	120.2
O3—C11—C3	110.2 (4)	C43—C44—C39	120.1 (4)
O3—C12—H12A	109.5	C43—C44—H44A	120.0
O3—C12—H12B	109.5	C39—C44—H44A	120.0
H12A—C12—H12B	109.5	C46—C45—C50	117.1 (4)
O3—C12—H12C	109.5	C46—C45—P2	124.7 (4)
H12A—C12—H12C	109.5	C50—C45—P2	118.0 (3)
H12B—C12—H12C	109.5	C47—C46—C45	121.6 (5)
O1—C13—O2	124.7 (5)	C47—C46—H46A	119.2
O1—C13—C8	123.3 (5)	C45—C46—H46A	119.2
O2—C13—C8	111.9 (5)	C48—C47—C46	120.1 (5)
O2—C14—H14A	109.5	C48—C47—H47A	120.0
O2—C14—H14B	109.5	C46—C47—H47A	120.0
H14A—C14—H14B	109.5	C47—C48—C49	118.8 (5)
O2—C14—H14C	109.5	C47—C48—H48A	120.6
H14A—C14—H14C	109.5	C49—C48—H48A	120.6
H14B—C14—H14C	109.5	C50—C49—C48	121.3 (5)
C20—C15—C16	118.9 (4)	C50—C49—H49A	119.3
C20—C15—P1	118.2 (4)	C48—C49—H49A	119.3
C16—C15—P1	122.8 (4)	C49—C50—C45	121.1 (5)
C17—C16—C15	120.8 (5)	C49—C50—H50A	119.5
C17—C16—H16A	119.6	C45—C50—H50A	119.5
C15—C16—H16A	119.6	C51—C52—H52A	109.5
C16—C17—C18	119.9 (5)	C51—C52—H52B	109.5
C16—C17—H17A	120.1	H52A—C52—H52B	109.5
C18—C17—H17A	120.1	C51—C52—H52C	109.5
C19—C18—C17	120.4 (5)	H52A—C52—H52C	109.5
C19—C18—H18A	119.8	H52B—C52—H52C	109.5
C17—C18—H18A	119.8		
